# Perceived Physical Fatigability: A Prognostic Marker of Biological, Organ System, and Brain Aging

**DOI:** 10.1093/geroni/igab046.795

**Published:** 2021-12-17

**Authors:** Nancy W Glynn, Eleanor Simonsick, Basil Eldadah

## Abstract

Characterizing perceived physical fatigability enables researchers to quantify an individual’s susceptibility to experiencing fatigue in the context of a standardized physical task. This approach eliminates self-pacing, and is a less-biased, more sensitive means to measure the degree to which fatigue may limit activity. Our previous work with two validated measures of perceived fatigability, the Pittsburgh Fatigability Scale (PFS) and Borg Rating of Perceived Exertion (RPE) at the end of a standardized 5-minute treadmill walk, are prognostic indicators of phenotypic aging. This symposium will present new directions related to greater fatigability as a marker of biological aging, organ system health and functioning, as well as brain pathology and structure. Specifically, Mr. Katz will explore the relationship between leukocyte telomere length, a marker of biological aging, with PFS fatigability in participants from the Long Life Family Study. The other four papers use data from the Baltimore Longitudinal Study of Aging (BLSA) and RPE fatigability (RPE). Drs. Simonsick and Karikkineth investigate fatigability as an early marker of aging and disease related impacts on key organ systems, specifically diminished renal function as reflected in estimated Glomerular Filtration Rate and cardiovascular health evaluated as vascular stiffness. Ms. Liu and Dr. Schrack will share whether there are associations of perceived fatigability with brain health, specifically Alzheimer’s disease-related pathology (PiB) and changes in brain structure. Lastly, our Discussant, Dr. Eldadah, will critically review the presentations in the context of new directions in fatigability research.

